# Ursolic acid derivative UA232 evokes apoptosis of lung cancer cells induced by endoplasmic reticulum stress

**DOI:** 10.1080/13880209.2020.1794013

**Published:** 2020-07-29

**Authors:** Wenfeng Gou, Na Luo, Huiqiang Wei, Hongying Wu, Xiaojun Yu, Yuqing Duan, Changfen Bi, Hongxin Ning, Wenbin Hou, Yiliang Li

**Affiliations:** Tianjin Key Laboratory of Radiation Medicine and Molecular Nuclear Medicine, Institute of Radiation Medicine, Peking Union Medical College & Chinese Academy of Medical Sciences, Tianjin, China

**Keywords:** Non-small cell lung cancer, endoplasmic reticulum stress

## Abstract

**Context:**

Ursolic acid (UA), a natural product, shows a broad spectrum of anticancer effects. However, the poor bioavailability and efficacy of UA limit its clinical application.

**Objective:**

We developed novel analogues of UA with enhanced antitumor activities by the extensive chemical modification of UA.

**Materials and methods:**

We developed multiple compounds by structural modification of UA, and found that UA232 had stronger activity than UA. The effects of UA232 (0–50 μM) on inhibiting the proliferation of A549 and H460 cells were determined by CCK-8 for 24, 48, or 72 h. The proapoptotic effect of UA232 was analyzed by microscopy and flow cytometry, and the potential signal pathway affected by UA232 was further validated by Western blotting and flow cytometry.

**Results:**

Compared with UA, UA232 showed a stronger ability to inhibit the proliferation of lung cancer cells (IC_50_ = 5.4–6.1 μM for A549 and 3.9–5.7 μM for H460 cells). UA232 could induce not only cell cycle arrest in the G0/G1 phase but also apoptosis in both A549 and H460 cells. The treatment of UA232 could lead to an increase of CHOP expression rather than an increase in Bax or caspase-8, indicating that the apoptosis induced by UA232 was correlated with the endoplasmic reticulum stress (ER stress) pathway. Treatment with the ER stress-specific inhibitor, 4-PBA, decreased the ability of UA232 to induce apoptosis in A549 and H460 cells.

**Conclusion:**

UA232 induced apoptosis through the ER stress pathway, and showed stronger growth-inhibitory effects in A549 and H460 cells compared to UA, which may be a potential anticancer drug to suppress the proliferation of lung cancer.

## Introduction

Lung cancer is the leading cause of cancer-related deaths worldwide (Buyukcelik et al. 2004). Non-small cell lung cancer (NSCLC), which accounts for 80–85% of lung cancer, is the most common histologic type of lung cancer and has the highest risk of lung cancer-related death (Jemal et al. 2011). Although there have been recent advances in treatment, the effect is not so inspiring because of the poor outcome (Shi et al. 2014). Therefore, it is necessary to develop a more effective treatment for lung cancer.

Over the years, around 60% of anticancer drugs approved by the FDA were natural products isolated from natural resources (Gupta et al. 2005). The structural modification of natural products can provide more anticancer clues and more effective candidate drugs (Grabley and Sattler 2003). Among various compounds obtained from natural resources, triterpenoids have become a research focus because of their good biological activity and functions suitable for chemical transformation (Mlala et al. 2019), which has attracted synthetic chemists and clinicians worldwide.

Ursolic acid (UA) is a pentacyclic triterpene complex that can be isolated from a variety of plants and fruits, such as rosemary, lavender, thyme, and apple peel (Chang et al. 2017). The early research of UA it was mainly used in antibacterial, antiviral, and immune regulation (Kim et al. 2013; do Nascimento et al. 2014). Multiple studies further demonstrated that UA had more pharmacological effects, such as blocking the process of chronic disease (Yu et al. 2017), radiosensitization (Yang et al. 2015), neuroprotective (Habtemariam 2019), antidiabetic (Xu et al. 2018), and antitumor (Zang et al. 2014). Studies have confirmed that UA plays an antitumor role in a variety of tumours, including colorectal cancer (Kim et al. 2018), breast cancer (Yin et al. 2018), endometrial carcinoma (Achiwa et al. 2013), and lung cancer (Yang et al. 2019). UA plays an antitumor role in a variety of ways, including inhibition of proliferation, angiogenesis, invasion, metastasis, differentiation, and induction of tumour cell apoptosis (Zhang et al. 2014). However, the poor bioavailability and efficacy of UA limit its clinical application in the treatment of lung cancer (Biswas et al. 2019).

To increase the bioavailability and antitumor effect of UA, we develop multiple new compounds by structural modification of UA. By analysing the growth-inhibitory effects of these compounds, we further identify a lead compound, UA232, with a stronger activity compared to UA. Our results provide a basis for developing UA232 as an effective candidate for the treatment of human non-small cell lung cancer.

## Materials and methods

### Chemical synthesis materials

Reactions were monitored by thin-layer chromatography (TLC) using silica gel 60 UV254 pre-coated silica gel plates. The detection was using a UV lamp. Flash column chromatography was performed on 300–400 mesh silica gel. ^1^H-NMR was recorded on a BRUKER AV400 spectrometer (Bruker Ltd., Faellanden, Switzerland) at 400 MHz. Chemical shifts were reported in ppm using CDCl3 solution with TMS as internal standards. Mass spectra (ESI-MS) were performed on Agilent 1200 HPLC-6310 liquid chromatography-mass spectrometer (Agilent Ltd., Palo Alto, CA). All reagents and solvents were used as received from commercial sources without further purification.

### (4-Bromo-butyl) 3-hydroxy-urs-12-en-28-oate (UA230)

To a solution of ursolic acid (5.00 g, 10.95 mmol, 1 eq.), K_2_CO_3_ (3.02 g, 21.90 mmol, 2 eq.), and KI (0.91 g, 5.48 mmol, 0.5 eq.) in 50 mL DMF, the mixture was stirred at room temperature. 1, 4-Dibromo-butane (4.73 g, 21.90 mmol, 2 eq.) was added dropwise to the mixture in 0.5 h and the reaction was monitored by TLC. When reactant was consumed completely, the mixture was poured into 300 mL water with stirring for 0.5 h and the mixture was stirred at 80 °C for 0.5 h and then was extracted with 300 mL ethyl acetate. The organic layer was dried with anhydrous Na_2_SO_4_, and then evaporated the solvent. The residue was purified by silica gel column chromatography (hexane: ethyl acetate = 15:1→8:1) to obtain 5.31 g UA-2-3-0 as white foamy solid with the yield of 81.9%. mp: 91.2–93.0 °C; ESI-MS *m*/*z* 591.34, 593.33 [M + H]^+^, ^1^H-NMR (400 MHz, CDCl_3_) *δ* 5.24 (t, *J* = 3.4 Hz, 1H), 4.02 (m, 2H), 1.75 (td, *J*1 = 13.6 Hz, *J*2 = 4.4 Hz, 1H), 1.67 (m, 1H), 1.64 (m, 2H), 1.62–1.57 (m, 3H), 1.54–1.45 (m, 4H), 1.40–1.27 (m, 4H), 1.06 (m, 5H), 0.99 (m, 1H), 0.85 (m, 9H), 0.82 (d, *J* = 11.6 Hz, 1H), 0.73 (s, 3H).

### [4-(Piperazin-1-yl)butyl] 3-hydroxy-urs-12-en-28-oate (UA232)

To a solution of UA-2-3-0 (1.00 g, 1.69 mmol, 1 eq.) and K_2_CO_3_ (0.47 g, 3.38 mmol, 2 eq.) in 15 mL DMF, the mixture was stirred at 80 °C for 0.5 h and then piperazine (0.44 g, 3.38 mmol, 2 eq.) was added. The reaction was monitored by TLC. When UA-2-3-0 consumed completely, the mixture was cooled to room temperature and then poured into 100 mL water with stirring for 0.5 h. Filtrated and dissolved the precipitate with ethyl acetate, then dried with anhydrous Na_2_SO_4_, 0.82 g UA-2-3-2 was obtained as a white solid powder after evaporating the solvent under reduced pressure with the yield of 81%. mp: 124.1–124.9 °C; ESI-MS *m*/*z* 597.43 [M + H]^+^; ^1^H-NMR (400 MHz, CDCl_3_) *δ* 5.22 (t, *J* = 3.2 Hz, 1H), 3.99 (m, 2H), 3.28 (s, 1H), 3.20 (m, 3H), 2.71 (s, 3H), 2.42 (m, 3H), 2.21 (d, *J* = 11.3 Hz, 1H), 1.99 (td, *J*1 = 13.2 Hz, *J*2 = 4.3 Hz, 1H), 1.87 (m, 2H), 1.75 (td, *J*1 = 13.7 Hz, *J*2 = 4.3 Hz, 1H), 1.56 (m, 14H), 1.30 (m, 5H), 1.07 (s, 3H), 1.04 (s, 1H), 0.97 (d, *J* = 6.1 Hz, 4H), 0.94 (s, 3H), 0.90 (s, 2H), 0.85 (d, *J* = 6.4 Hz, 3H), 0.77 (s, 3H), 0.73 (s, 3H), 0.71 (d, *J* = 12.5 Hz, 1H).

### Bioassay materials

The purity of UA232 was more than >98%. UA was purchased from MedChemExpress (Monmouth Junction, NJ). UA232 and UA were dissolved in dimethyl sulfoxide (DMSO). Crystal violet was purchased from Beyotime Biotechnology (Shanghai, China). Polyvinylidenedifluoride (PVDF) membrane was purchased from Millipore Bedford (Bedford, MA). Propidium iodide (PI), FITC-labeled Annexin V, and propidium iodide (PI) double-staining were purchased from KeyGen Biotech (Nanjing, China). Primary antibodies against cyclin D1, CDK4, Bax, Bcl-2, caspase-8, PARP1, and CHOP were purchased from Proteintech Group (Rosemont, IL). Antibodies for PERK, p-PERK, eIF2α, and p-eIF2α were purchased from Bioss (Beijing, China). β-actin and horseradish peroxidase (HRP)-conjugated secondary antibodies (goat-anti-mouse or goat-anti-rabbit) were obtained from ZSGB-BIO (Beijing, China).

### Cell culture

Human non-small cell lung cancer cell lines H460, A549, and human embryonic kidney cell line HEK293T were obtained from the American Type Culture Collection (ATCC). The A549 and H460 cells were cultured in RPMI 1640 medium supplemented with 10% foetal bovine serum (FBS), 1% penicillin, and 1% streptomycin. The HEK293T cells were cultured in DMEM medium, other medium components were consistent with other cells. All the cells were incubated at 37 °C with 5% CO_2_.

### CCK8 assay

A549, H460, or HEK293T cells were seeded in 96-well plates at a density of 3000 cells/well and cultured for 24 h. Treatment cells with different concentrations of UA232 or UA for 24, 48, or 72 h. Then 20 μL CCK8 was added to each well and incubated at 37 °C for 2 h. Optical density at 450 nm was measured using a TECAN Infinite 200 (Männedorf, Switzerland).

### Colony formation assay

A549 or H460 cells were seeded into 6-well plates at a density of 5000 cells/well and incubated for 24 h. Treatment cells with different concentrations of UA232 or UA for 48 h and then remove it with fresh medium. After 2 weeks of growth, the cells were fixed at room temperature with 4% paraformaldehyde for 30 min and stained with crystal purple for 15 min. The colonies were photographed by Sony digital camera.

### Cell cycle analysis

A549 or H460 cells were seeded into 25 cm^2^ cell culture bottle at a density of 3 × 10^5^ cells/bottle and incubated for 24 h. Treatment of cells with UA232 for 24, 48, or 72 h. The collected cells were fixed with pre-cooled 70% ethanol for 24 h, then washed with PBS twice and stained with PI working solution containing RNase at room temperature for 30 min. Then the samples were analyzed by FACScan flow cytometry (Becton-Dickinson, Franklin Lake, NJ). The distribution of the cell cycle was analyzed by ModFit LT software.

### Apoptosis analysis

A549 or H460 cells were seeded into 25 cm^2^ cell culture bottle at a density of 3 × 10^5^ cells/bottle and incubated for 24 h. Cells were treated with different concentrations of UA232 or UA for 48 h, then collected and stained using an Annexin V-FITC/PI staining kit. Samples were analyzed by FACScan flow cytometry (Becton-Dickinson, Franklin Lakes, NJ). Apoptosis rate analysis was done using ImageJ2 software.

### Western blotting

A549 or H460 cells were seeded into 25 cm^2^ cell culture bottle at a density of 3 × 10^5^ cells/bottle and incubated for 24 h. Cells were treated with different concentrations of UA232 or UA for 48 h. Cells were collected and lysed in a RIPA buffer at 4 °C for 1 h. After centrifugation at 14,000 rpm for 20 min, the protein concentration of the supernatant was determined using a BCA Protein Assay Kit (Beyotime Biotechnology, Shanghai, China). Protein samples (40 μg) were electrophoresis on 10% SDS-polyacrylamide gel and then transferred to PVDF membrane. The membranes were incubated with 5% skimmed milk at room temperature for 1 h, then incubated overnight with a specific primary antibody for 4 °C. On the second day, the membrane was incubated with the corresponding HRP-conjugated secondary antibody (1:3,000) for 2 h. The target protein was detected by the chemiluminescence instrument. The grey value of the western blot was analyzed by ScnImage software.

### Statistical analysis

Statistical differences between groups were assessed by a one-way ANOVA using SPSS software 22.0. *p* < 0.05 was considered statistically significant. Data were expressed as mean ± S.D.

## Results

### UA232 inhibits the growth of lung cancer cells

**Figure 1.**
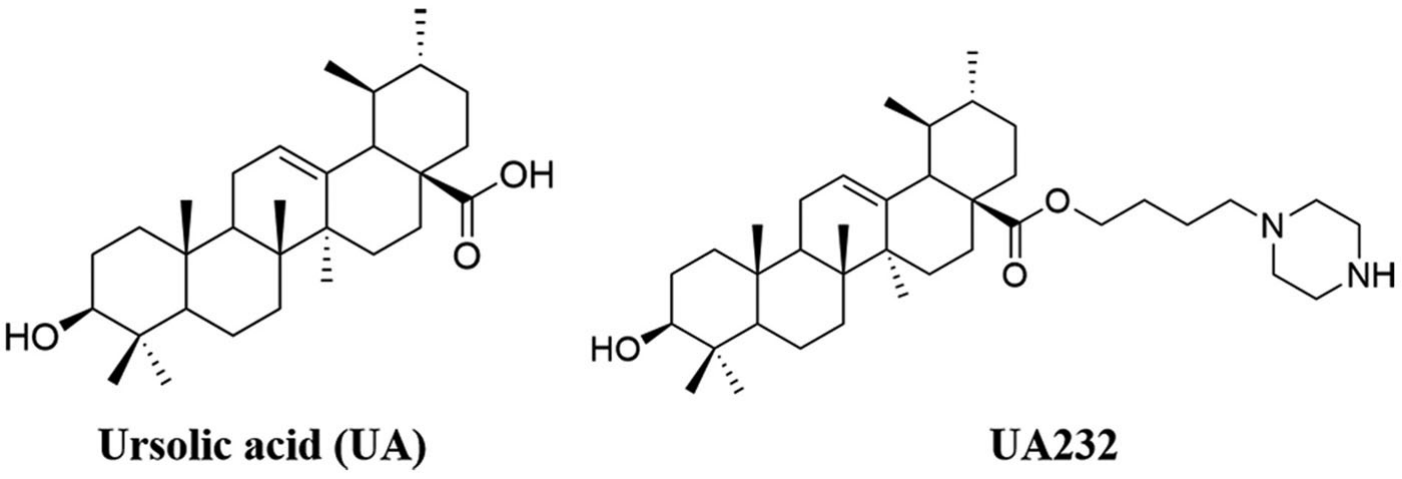
The chemical structures of ursolic acid (UA) and UA232.

**Figure 2.**
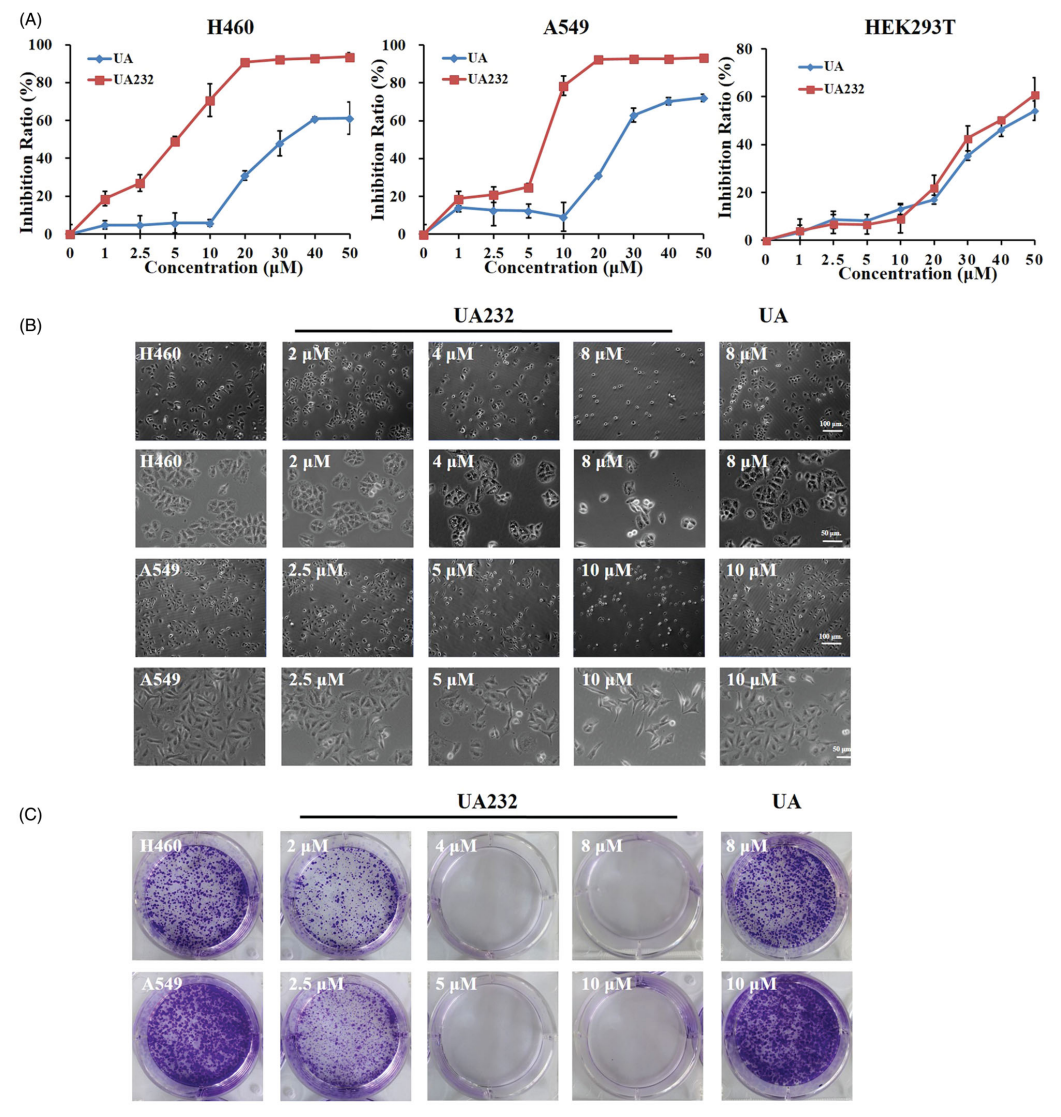
Effects of UA232 on H460 and A549 cell viability and proliferation. (A) The cell inhibition rate was determined by CCK8 assay in H460 and A549 cells treated with UA232 or UA (0–50 μM) for 72 h. (B) Cellular morphology of H460 and A549 cells after treatment with different concentrations of UA232 or UA for 48 h. (C) Effect of UA232 or UA on colony formation. The experiment was repeated at least three times.

In the early stage, we synthesized a series of compounds by structural modification of UA, and found that compared with UA, the novel compound UA232 has a more significant inhibitory effect on the cell viability of A549 and H460 cells. The chemical structures and characterization of UA232 were shown in Figure 1 and Supplemental Figure 1. Compared with UA, UA232 exhibited stronger cytotoxicity against H460 and A549 cells, while the cytotoxicity of UA232 to human normal cell HEK293T was similar to that of UA (Figure 2(A)). Table 1 shows the IC_50_ values with UA232 for H460 and A549 cells at 24, 48, and 72 h. The inhibitory effect of UA232 on cell viability was significantly stronger than that of UA at each time point. As shown in Figure 2(B), cell growth of H460 and A549 was dramatically inhibited by the treatment of UA232, as indicated by the decrease of cell density compared to the control group. Moreover, the treatment of UA233 caused vacuole-like changes in the morphology of H460 and A549 cells (Figure 2(B)). At 8 µM, most of the cells lost their morphology, and the cell membrane was incomplete (Figure 2(B)). Colony formation assay was carried out to verify the inhibitory effect of UA232 on the proliferation of lung cancer cells. Figure 2(C) shows that UA232 dose dependently inhibited the colony formation of H460 and A549 cells.

**Table 1.** The IC_50_ values of UA232 and UA by CCK8 assay.

	H460 (IC_50_ μM)			A549 (IC_50_ μM)		
	24 h	48 h	72 h	24 h	48 h	72 h
UA	40.7 ± 3.8	35.1 ± 2.5	28.9 ± 1.3	34.4 ± 2.4	30.4 ± 1.5	26.8 ± 2.3
UA232	5.7 ± 1.1	4.5 ± 0.4	3.9 ± 0.2	6.1 ± 1.1	5.5 ± 1.2	5.4 ± 0.5

### UA232 induces G0/G1 phase arrest in A549 and H460 cells

**Figure 3.**
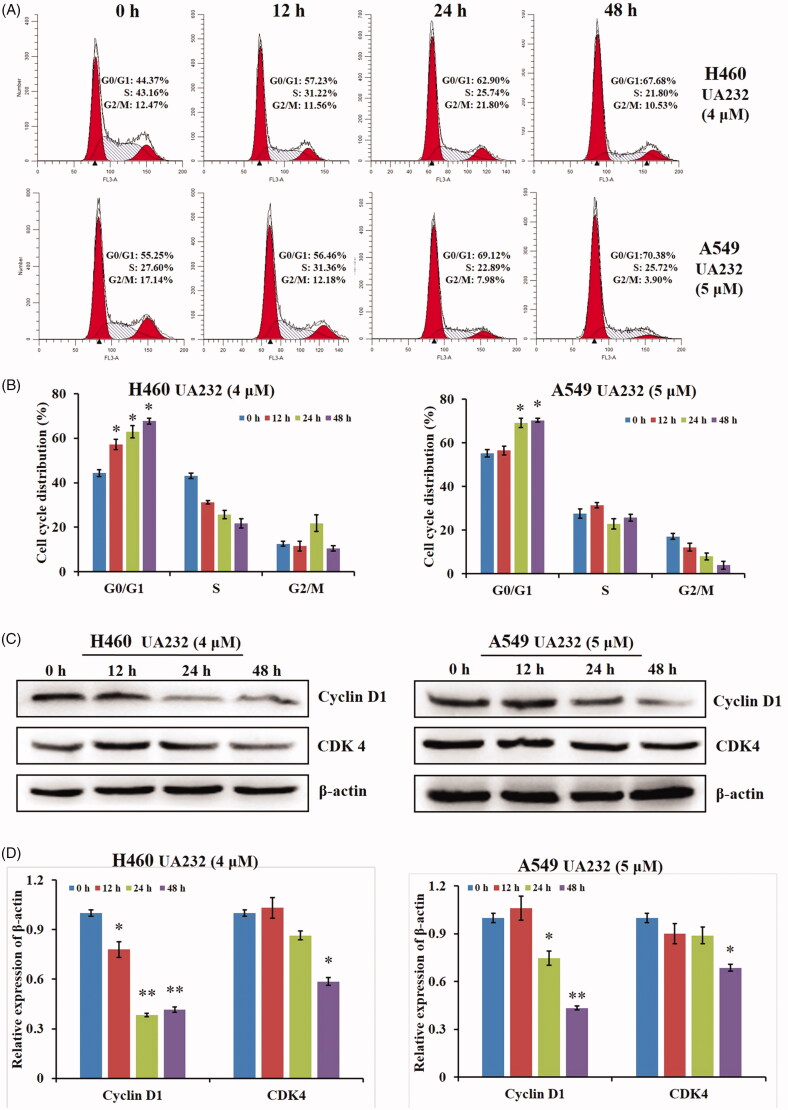
UA232 arrests the cell cycle in H460 and A549 cells. (A) The cell cycle distribution of H460 or A549 cells treated with UA232 (4 or 5 μM) for the indicated time (0, 24, 48, or 72 h) was measured by flow cytometry with PI staining. (B) The percentage of cells in specific cell cycle phase was quantified in panel A. (C) The expression of G0/G1 phase-related protein in H460 or A549 cells treated with UA232 (4 or 5 μM) for 0–72 h was analysed by western blot. (D) The quantitative data of western blot analysis in panel C. *p < 0.05 versus control, **p < 0.01 versus control.

The effects of UA232 on the cell cycle regulation of H460 or A549 cells were further analyzed by flow cytometry. As shown in Figure 3(A,B), UA232 (4 or 5 μM) arrested the H460 or A549 cells in the G0/G1 phase. To further explore the mechanism G0/G1 phase arrest induced by UA232, we examined the expression of G0/G1 phase-related proteins, cyclin D1 and CDK4. Figure 3(C,D) shows that UA232 (4 μM) significantly down-regulated cyclin D1 and CDK4 during 24–72 h in H460 and A549 cells. These data suggested that UA232 could induce cell cycle arrest in the G0/G1 phase and down-regulation of cyclin D1 and CDK4 in H460 and A549 cells.

### UA232 induces apoptosis in A549 and H460 cells

**Figure 4.**
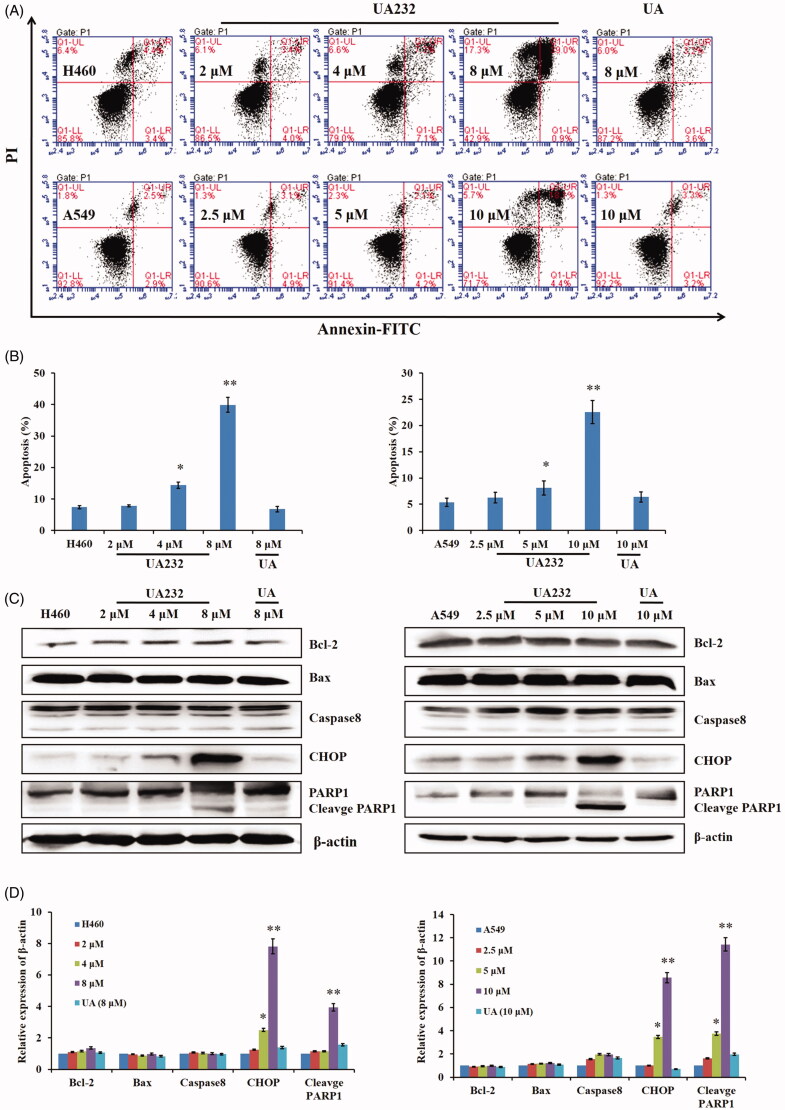
UA232 induces apoptosis in H460 and A549 cells. (A) The apoptosis of H460 and A549 cells treated with different concentrations of UA232 or UA for 48 h were measured by flow cytometry with Annexin V-FITC staining. (B) The percentage of apoptosis rate in H460 and A549 cells was quantified in panel A. (C) Changes of the expression of cell apoptosis-related proteins in H460 and A549 cells treated with different concentrations of UA232 or UA for 48 h. (D) The quantitative data of Western blot analysis in panel C. *p < 0.05 versus control, **p < 0.01 versus control.

We further applied Annexin V-FITC/PI staining analysis to verify whether UA232 could induce apoptosis in H460 and A549 cells. As shown by the flow cytometry analyses in Figure 4(A,B), UA232 induced apoptosis in H460 and A549 cells, and the apoptosis rate of H460 and A549 cells increased with the increase of UA232 concentration. Under the same concentration, the pro-apoptotic effect of UA232 was stronger than that of UA (Figure 4(A,B)). A variety of crucial pathways regulate cell apoptosis, including the intrinsic pathway of mitochondrial apoptosis (Liu and Fan 2019), the extrinsic pathway of death receptor (Mughal et al. 2017), and ER stress pathway (Zhang et al. 2017). In order to explore which pathway of apoptosis induced by UA232, we detected the expression of mitochondrial apoptosis pathway marker protein Bcl-2 and Bax (Cory and Adams 2002), death receptor pathway marker protein caspase-8 (Ivanova et al. 2019), and ER stress pathway marker protein CHOP (Kim et al. 2017). As shown in Figure 4(C,D), UA232 increased the levels of CHOP and cleavage-PARP1 in H460 and A549 cells but had no significant effects on the levels of Bax, Bcl-2, and caspase-8. These data indicated that UA232 could induce apoptosis via the ER stress-related pathway in lung cancer cells.

### Apoptosis induced by UA232 is correlated with ER stress pathway

**Figure 5.**
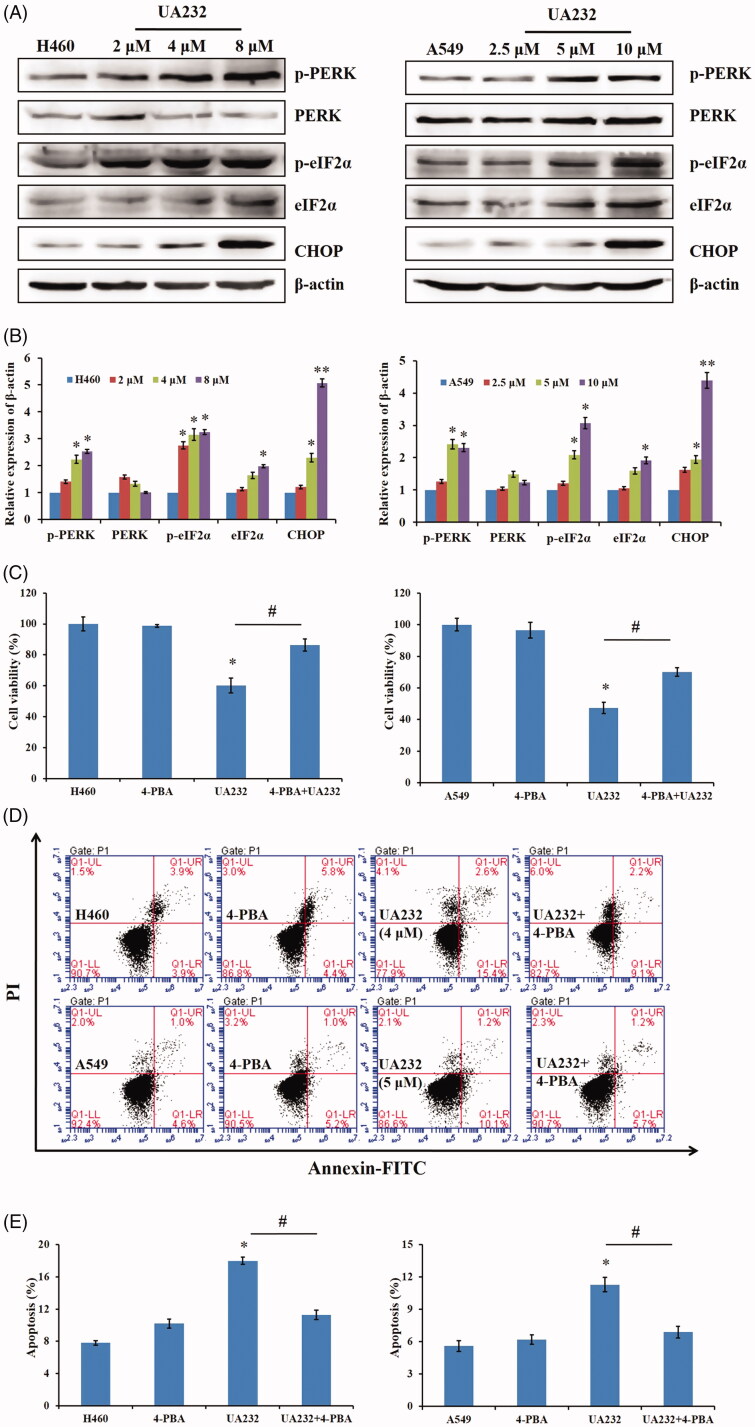
UA232 triggers ER stress leading to apoptosis. (A) Changes in the expression of cell ER stress-related proteins in H460 and A549 cells treated with different concentrations of UA232 for 48 h. (B) The quantitative data of Western blot analysis in panel A. (C) The cell viability of H460 and A549 cells treated with UA232 (4 or 5 μM), 4-PBA, or both for 48 h were measured by CCK8 assay. (D) The apoptosis of H460 and A549 cells treated with UA232 (4 or 5 μM), 4-PBA or both for 48 h were measured by flow cytometry with Annexin V-FITC staining. (E) The percentage of apoptosis rate in H460 and A549 cells was quantified in panel D. *p < 0.05 versus control, **p < 0.01 versus control, *^#^p* < 0.05 versus UA232.

ER stress-mediated apoptosis is associated with PERK/eIF2α-dependent induction of the pro-apoptotic transcriptional factor CHOP (Ghosh et al. 2012). As shown in Figure 5(A,B), the expression levels of p-PERK, p-eIF2α, and CHOP were increased significantly with the increased concentration of UA232 in H460 and A549 cells, indicating that UA232 induced ER stress in lung cancer cells. In order to explore the role of ER stress in UA232-induced H460 and A549 cells apoptosis, H460 or A549 cells were pre-treated with the ER stress inhibitor 4-PBA (2.5 mM) for 2 h and then treated with UA232 for 24 h. The CCK8 assay results showed that inhibition of ER stress by 4-PBA partly reversed the pro-apoptotic effect of UA232 (Figure 5(C)). Moreover, the flow cytometry results further proved that the combination of 4-PBA and UA232 reduced the apoptosis rate of H460 and A549 cells compared with UA232 alone (Figure 5(D,E)). These data suggested that UA232 induced ER stress, which participated in the process of UA232-induced cell proliferation decrease and cell apoptosis.

## Discussion

About 60% of approved anticancer drugs come from natural resources, so natural products play a leading role in drug discovery (Gupta et al. 2005). Triterpenes have been the focus of drug development because of their good transformation function, biological activity, and ubiquitous characteristics. UA, a pentacyclic triterpenoid acid, is found abundantly in plants, especially in fruit and vegetable (Chang et al. 2017). Although UA plays a variety of biological activities, its poor bioavailability seriously limits its clinical application (Biswas et al. 2019). Structural modification of bioactive natural products has become an important means of developing potential bioactive molecules and leading drugs (Grabley and Sattler 2003). We synthesized a series of compounds by modifying the structure of ursolic acid and found that UA232 has stronger antitumor activity.

In the present study, UA232 potently inhibited cell viability and colony formation ability of H460 and A549 cells. Interestingly, there was no significant difference in IC_50_ of UA232 at 24, 48, and 72 h, indicating that UA232 could exert its antitumor effect more quickly. Due to the high activity of UA232 on lung cancer cells, the cellular effects and molecular mechanisms of UA232 on H460 and A549 cells were further studied for the first time. Colony formation assays suggest that the growth of H460 and A549 cells has been inhibited by UA232. Therefore, the effects of UA232 on the cell cycle distribution in H460 and A549 cells were further investigated. Flow cytometry indicated that UA232 arrested the growth of H460 and A549 cells at the G0/G1 phase. It has been shown that inhibition of the expression of cell cycle-related proteins CDK4 and cyclin D1 can lead to cell cycle arrest in the G0/G1 phase and inhibit cell proliferation (Kim et al. 2005). We further confirmed that UA232 induced G0/G1 arrest by detecting the expression of CDK4 and cyclin D1.

The results of cell morphology observation showed that UA232 could lead to vacuole-like changes in lung cancer cells and lead to cell death. The results of Annexin V-FITC/PI double staining further confirmed that UA232 could induce apoptosis of H460 and A549 cells, indicating that UA232 induced cell death by inducing apoptosis. Apoptosis is regulated by a variety of pathways, including the death receptor pathway, mitochondrial apoptosis pathway, and endoplasmic reticulum stress pathway (Li et al. 2017; Cha et al. 2019; Lee et al. 2019). We found that the endoplasmic reticulum stress marker protein CHOP increased significantly, indicating that UA232 might induce apoptosis of lung cancer cells through the ER stress pathway.

CHOP is a marker of PERK branch activation in the ER stress pathway (Walter and Ron 2011). The activation of CHOP downstream of PERK signalling pathway plays an important role in ER stress-induced apoptosis of tumour cells (Hetz 2012; Rozpedek et al. 2016). Therefore, we further detected the changes of other proteins in the PERK pathway and found that the expression of p-PERK and p-eIF2α, the active forms of PERK and eIF2α, increased, which further confirmed our conjecture that UA232 promotes apoptosis through ER stress pathway. To further explore the role of ER stress in apoptosis induced by UA232, we blocked ER stress by 4-PBA and found that blocking ER stress could significantly reverse UA232-induced apoptosis in H460 and A549 cells.

## Conclusions

UA232 is a novel UA analogue, which exhibits a potent antitumor effect *in vitro*. UA232 inhibited cell proliferation by inducing the G0/G1 phase arrest, and induced cell apoptosis, which is correlated with the ER stress pathway, especially the activation of the PERK/eIF2α/CHOP axis. To sum up, the novel compound UA232 is a promising anticancer drug.
